# Network-Based Expression Analyses and Experimental Verifications Reveal the Involvement of STUB1 in Acute Kidney Injury

**DOI:** 10.3389/fmolb.2021.655361

**Published:** 2021-06-28

**Authors:** Yanting Shi, Genwen Chen, Jie Teng

**Affiliations:** ^1^ Department of Nephrology, Xiamen Branch, Zhongshan Hospital, Fudan University, Shanghai, China; ^2^ Department of Radiation Oncology, Zhongshan Hospital, Fudan University, Shanghai, China; ^3^ Department of Nephrology, Zhongshan Hospital, Fudan University, Shanghai, China

**Keywords:** acute kidney injury, cisplatin, STUB1, RNA sequencing, network-based expression analysis

## Abstract

Acute kidney injury (AKI) is a severe and frequently observed condition associated with high morbidity and mortality. The molecular mechanisms underlying AKI have not been elucidated due to the complexity of the pathophysiological processes. Thus, we investigated the key biological molecules contributing to AKI based on the transcriptome profile. We analyzed the RNA sequencing data from 39 native human renal biopsy samples and 9 reference nephrectomies from the Gene Expression Omnibus (GEO) database. The differentially expressed genes (DEGs) and Gene Ontology (GO) analysis revealed that various GO terms were dysregulated in AKI. Gene set enrichment analysis (GSEA) highlighted dysregulated pathways, including “DNA replication,” “chemokine signaling pathway,” and “metabolic pathways.” Furthermore, the protein-to-protein interaction (PPI) networks of the DEGs were constructed, and the hub genes were identified using Cytoscape. Moreover, weighted gene co-expression network analysis (WGCNA) was performed to validate the DEGs in AKI-related modules. Subsequently, the upregulated hub genes STUB1, SOCS1, and VHL were validated as upregulated in human AKI and a mouse cisplatin-induced AKI model. Moreover, the biological functions of STUB1 were investigated in renal tubular epithelial cells. Cisplatin treatment increased STUB1 expression in a dose-dependent manner at both the mRNA and protein levels. Knockdown of STUB1 by siRNA increased the expression of proapoptotic Bax and cleaved caspase-3 while decreasing antiapoptotic Bcl-2. In addition, silencing STUB1 increased the apoptosis of HK-2 cells and the proinflammatory cytokine production of *IL6*, *TNFα*, and *IL1β* induced by cisplatin. These results indicated that STUB1 may contribute to the initiation and progression of AKI by inducing renal tubular epithelial cell apoptosis and renal inflammation.

## Introduction

Acute kidney injury is characterized by the abrupt onset of abnormal kidney functions. As a common clinical symptom, AKI is defined by a surge in serum creatinine and a rapid decrease in urine output within a short time (Khwaja, 2012). In recent decades, the incidence of AKI has steadily increased. The incidence of AKI in ordinary hospitalized patients is 5–20%, and the incidence in intensive care units is as high as 30–60% (Jacob et al., 2020). AKI is induced by various conditions, such as suffering from ischemia, toxic agents, decreased kidney perfusion, and inflammation (Ali et al., 2007). Although many factors are known to be responsible for AKI, the underlying molecular mechanisms are still poorly understood.

Emerging evidence has indicated that various potential mechanisms are involved in AKI. Recently, different cell deaths, such as necrotic cell death, proptosis, and ferroptosis, iron-dependent form of non-apoptotic cell death, were highlighted in AKI (Linkermann et al., 2014). In response to hypoxia, changes in mitochondria and oxidative stress also play vital roles in cell death through mitochondrial permeability transition (MPT) and reactive oxygen species (ROS) (Zamzami and Kroemer, 2001). Furthermore, PGC1α-mediated mitochondrial biogenesis also influences the recovery of ischemia–reperfusion injury (Funk and Schnellmann, 2012). Cell cycle–related proteins, the AP-1 family, NF-κB, HIF, and Nrf2 have been identified to regulate AKI *via* regulation of various genes. In addition, the PERK-ATF4-CHOP pathway mediates endoplasmic reticulum (ER) stress (Taniguchi and Yoshida, 2015), autophagy (Kaushal and Shah, 2016), the TGFβ receptor (Linkermann et al., 2014), the HGF receptor (c-Met) (Zhou et al., 2013), p53 signaling (Zhang et al., 2014), etc., and these functions are associated with the initiation and development of AKI. Collectively, these results suggest that the molecular mechanisms of AKI are comprehensive. Hence, identifying potential molecular targets responsible for AKI will tremendously benefit patients suffering from AKI.

Next-generation sequencing (NGS) has been widely used to reveal the potential biomarkers and molecular mechanisms of AKI. In ischemia–reperfusion (I/R) kidney injury, RNA-seq from five pairs of human normal and I/R kidney tissues revealed that apoptosis-, metabolism-, and fibrosis-related pathways were dysregulated in I/R tissues (Park et al., 2020). Another cohort of RNA sequencing data found that apoptosis, migration, oxidation–reduction, and proliferation of macrophages were involved in I/R injury. Changes in CD11b+/Ly6c macrophages and polarization participated in the process of AKI (Sun et al., 2018). In an I/R AKI mouse model, transcriptome sequencing of renal tubules suggested that metabolic maladaptation was implicated in AKI progression to chronic fibrosis, with massive lipid droplet accumulations observed in renal tubular epithelial cells (Zhang et al., 2018). In cisplatin-induced acute kidney injury, methylated RNA immunoprecipitation sequencing and RNA sequencing revealed that metabolic processes and cell death were activated in the injury group (Shen et al., 2020). In mouse sepsis-associated AKI, the differentially expressed genes (DEGs) from three paired tissues’ RNA-seq were enriched in the nod-like receptor signaling, toll-like receptor signaling, and JAK/STAT signaling pathways (Yang et al., 2020). However, due to the limited availability of human AKI samples in the clinic, few studies have investigated the molecular mechanism of AKI based on clinical practice.

In the present study, we investigated gene expression profiles from RNA-seq data in GEO datasets (GSE139061) from 39 native human AKI renal biopsy samples and 9 reference nephrectomies. Subsequently, bioinformatic analyses were performed to assess the DEGs and potential key molecules responsible for AKI. Moreover, we then validated the upregulated DEGs from human renal biopsy samples and a cisplatin-induced kidney injury model. The biological function of the novel molecule *STUB1* (STIP1 homology and U-Box containing protein 1) was further investigated in human proximal tubular cells. Further investigations are warranted to reveal the mechanisms of the novel discovered signatures related to AKI.

## Materials and Methods

### Data Processing and Identification of Differentially Expressed Genes

Gene expression analysis was performed using the RNA sequencing profile GSE139061 from the Gene Expression Omnibus (GEO) database. The count values were regularized *via* log transformation using the DESeq2 R package. Protein-coding genes were included, while genes with low expression were excluded from further analysis. Significant DEGs were defined at a cutoff value of |log2 fold change|≥1 and *p*-value < 0.05. The enriched pathways were determined using the identified DEGs. The GEO dataset was analyzed using the R statistical environment, version 4.0.1.

### Gene Set Enrichment Analysis

GSEA was performed using GSEA software (v4.0.3, www.broadinstitute.org/gsea). The statistical significance and enrichment degree were quantified by the nominal *p*-value and normalized enrichment score (NES). Kyoto Encyclopedia of Genes and Genomes (KEGG) pathways were used to evaluate enriched pathways by GSEA software.

### Gene Ontology (GO) Analysis

GO analysis was performed to analyze the enriched biological themes of DEGs for the classifications of biological process (BP), cellular component (CC), and molecular function (MF).

### PPI Network Construction

The Search Tool for the Retrieval of Interaction Genes/Proteins (STRING v11.0, www.string-db.org) database was used to construct the protein–protein interaction (PPI) network of DEGs. Subsequently, Cytoscape 3.8.0 was utilized to plot network diagrams, and the hub genes were identified by the cytoHubba plugin.

### Weighted Gene Co-Expression Network Analysis (WGCNA)

The WGCNA R package was performed to construct a scale-free network. The co-expression network was achieved by setting the soft-thresholding power as 8 according to the scale-free topology criterion with a correlation coefficient of 0.9. The highly correlated genes were divided into gene modules using cluster analysis. The gene module size less than 30 was excluded. We defined the cut height threshold as 0.25 to explore the gene modules. The gray module represented background genes that belonged to none of the modules.

### Patients

Eleven patients (five AKI and six normal) scheduled for kidney biopsy because of kidney malfunction were enrolled in this study. The kidney tissues were obtained by gun biopsy. Pathological injury of the kidneys was assessed in a blinded fashion by two independent pathologists. Our study was approved by the Ethics Committee of Xiamen Branch, Zhongshan Hospital, Fudan University. All the participants signed informed consent forms, and the procedures were implemented according to the Helsinki Declaration.

### Mouse Cisplatin-Induced Acute Kidney Injury Model

Eight-week-old female C57BL/6J mice (20 g) were housed under conditions of 40–70% humidity and 22 ± 2°C with a 12 h light/dark cycle and free access to adequate food and water. A total of 12 mice were randomly divided into two groups: control and 20 mg/kg cisplatin. Subsequently, the mice were intraperitoneally (i.p.) injected with cisplatin at the indicated concentration or equivalent volume of sterile 0.9% saline. 72 h after injection, the mice were sacrificed, and the kidney and serum samples were collected for further analysis. The study protocol was approved by our institutional committee. All protocols used in this study were conducted according to the Guide for the Care and Use of Laboratory Animals of Zhongshan Hospital, Fudan University, based on the Helsinki Declaration.

### Serum Creatinine Assessment

Blood samples were centrifuged at 3,000 g for 15 min at room temperature to collect the supernatants. The serum creatinine (Scr) levels were measured using Creatinine Assay Kit (QuantiChrom™) according to the manufacturer’s instructions.

### Cell Culture and Treatment

The human proximal tubular cell line HK-2 was purchased from American Type Culture Collection (ATCC) and cultured in Dulbecco’s Modified Eagle Medium (DMEM)/Nutrient Mixture F-12 medium supplemented with 10% heat-inactivated fetal bovine serum (FBS, Biological Industries, Cromwell, CT, United States) and 1% penicillin–streptomycin (Gibco, MA, United States) in a humidified incubator with 5% CO_2_ at 37°C. The cells were treated with cisplatin (MedChemExpress, United States) at the indicated concentration for 24 and 48 h and subsequently harvested to determine the mRNA and protein levels.

### RNA Interference

siRNAs were transfected into HK-2 cells with Lipofectamine 3000 (Thermo Fisher Scientific, Pittsburgh, PA, United States) according to the manufacturer’s instructions. Specific siRNAs were synthesized by RiboBio (Guangzhou, China), and the sequences are as follows: siSTUB1#1 (5′-AAC​AGG​CAC​UUG​CUG​ACU​GTT-3′), siSTUB1#2 (5′- AGG​CCA​AGC​ACG​ACA​AGU​A-3′). The knockdown efficiency was determined by qRT-PCR and western blot.

### Quantitative Real-Time PCR

Total RNA from cells or tissues was extracted with TRIzol reagent (Takara Bio, Shiga, Japan), and then 500 ng RNA was reverse transcribed into cDNA by PrimeScript RT reagent Kit with gDNA Remover (Takara). qRT-PCR was performed using SYBR Premix Ex Taq II (TaKaRa) on a QuantStudio 5 machine (Thermo Fisher Scientific). The relative gene expression was normalized to GAPDH using the 2^-∆∆Ct^ method. The primer sequences used for qRT-PCR are as follows: human *GAPDH* (forward: 5′-GGA​GCG​AGA​TCC​CTC​CAA​AAT-3'; reverse: 5′-GGC​TGT​TGT​CAT​ACT​TCT​CAT​GG-3′), human *STUB1* (forward: 5′-AGC​AGG​GCA​ATC​GTC​TGT​TC-3'; reverse: 5′-CAA​GGC​CCG​GTT​GGT​GTA​ATA-3′), human *VHL* (forward: 5′-GCA​GGC​GTC​GAA​GAG​TAC​G-3'; reverse: 5′-CGG​ACT​GCG​ATT​GCA​GAA​GA-3′), human *SOCS1* (forward: 5′-CAC​GCA​CTT​CCG​CAC​ATT​C-3'; reverse: 5′-TAA​GGG​CGA​AAA​AGC​AGT​TCC-3′), human *IL6* (forward: 5′-ACT​CAC​CTC​TTC​AGA​ACG​AAT​TG-3'; reverse: 5′-CCA​TCT​TTG​GAA​GGT​TCA​GGT​TG-3′), human *TNFα* (forward: 5′-CCT​CTC​TCT​AAT​CAG​CCC​TCT​G-3'; reverse: 5′-GAG​GAC​CTG​GGA​GTA​GAT​GAG-3′), human *IL1β* (forward: 5′-ATG​ATG​GCT​TAT​TAC​AGT​GGC​AA-3'; reverse: 5′-GTC​GGA​GAT​TCG​TAG​CTG​GA-3′), mouse *GAPDH* (forward: 5′-AGG​TCG​GTG​TGA​ACG​GAT​TTG-3'; reverse: 5′-TGT​AGA​CCA​TGT​AGT​TGA​GGT​CA-3′), mouse STUB1 (forward: 5′-CGG​CAG​CCC​TGA​TAA​GAG​C-3'; reverse: 5′-CAC​AAG​TGG​GTT​CCG​AGT​GAT-3′), mouse *VHL* (forward: 5′-CTC​AGC​CCT​ACC​CGA​TCT​TAC-3'; reverse: 5′-ACA​TTG​AGG​GAT​GGC​ACA​AAC-3′), and mouse *SOCS1* (forward: 5′-CTG​CGG​CTT​CTA​TTG​GGG​AC-3'; reverse: 5′-AAA​AGG​CAG​TCG​AAG​GTC​TCG-3′).

### Western Blot

HK-2 cells or animal kidney tissue lysates were prepared in RIPA buffer (Beyotime Biotechnology, Shanghai, China). The protein concentrations were detected by the BCA method (Beyotime). Briefly, 40 μg of protein was subjected to 10% sodium dodecyl sulfate–polyacrylamide gel electrophoresis (SDS-PAGE). Then, the proteins were transferred to a polyvinylidene fluoride (PVDF) membrane (Millipore, Billerica, MA, United States). After blocking with 5% non-fatty milk for 1 h at room temperature, the membranes were incubated with primary antibodies of STUB1 (Proteintech, Wuhan, China), VHL (Proteintech), SOCS1 (Proteintech), tubulin (Proteintech), Bcl-2 (Cell Signaling, Beverly, MA, United States), Bax (Cell Signaling), cleaved caspase-3 (Cell Signaling), and caspase-3 (Cell Signaling) at 4°C overnight. Then, the membranes were incubated with HRP-conjugated anti-rabbit secondary antibody (Cell Signaling) at room temperature for 1 h. The proteins were then visualized by incubating with a Chemiluminescence (ECL) Western Blot Kit (Thermo Fisher Scientific).

### Histology and Immunohistochemistry

The hemisected kidneys were fixed with 10% formalin and then embedded in paraffin wax. After cutting into 4 μm-thick sections, the slides were stained with hematoxylin and eosin (HE) and evaluated under a light microscope. For immunohistochemistry (IHC) staining, antigen retrieval was performed after deparaffinization. Subsequently, endogenous peroxidase activity was blocked by 3% H_2_O_2_ and then incubated with 5% BSA for 30 min at 37°C. The sections were incubated with primary antibodies against STUB1 (Proteintech), VHL (Proteintech), and SOCS1 (Proteintech) overnight. Then, after incubation with HRP-conjugated secondary antibody, the proteins were visualized using DAB (Beyotime).

### Annexin V/PI Cell Apoptosis Assay

Apoptosis of HK-2 cells was quantified using flow cytometry. The HK-2 cells were transfected with 50 nM siRNA targeting STUB1 or control. After 24 h, the HK-2 cells were treated with 20 μM cisplatin for 24 h. Subsequently, the cells were trypsinized down and washed with cold PBS. The cells were then resuspended with Annexin V and PI (BD Biosciences, San Jose, CA, United States) according to the manufacturer’s instructions. The apoptosis cells were determined using a BD flow cytometer and analyzed using FlowJo10 (FlowJo LLC, Ashland, OR, United States).

### Statistical Analysis

The data are presented as mean ± standard deviation (SD). Student’s *t*-test and one-way analysis of variance (ANOVA) were used to compare data between two groups and multiple groups, respectively. The experiments were performed with at least three biological replicates. The statistical analyses were performed in SPSS 22.0 (SPSS Inc., Chicago, IL), and *p* values less than 0.05 were considered statistically significant.

## Results

### Dysregulated Gene Identification in AKI

**FIGURE 1:**
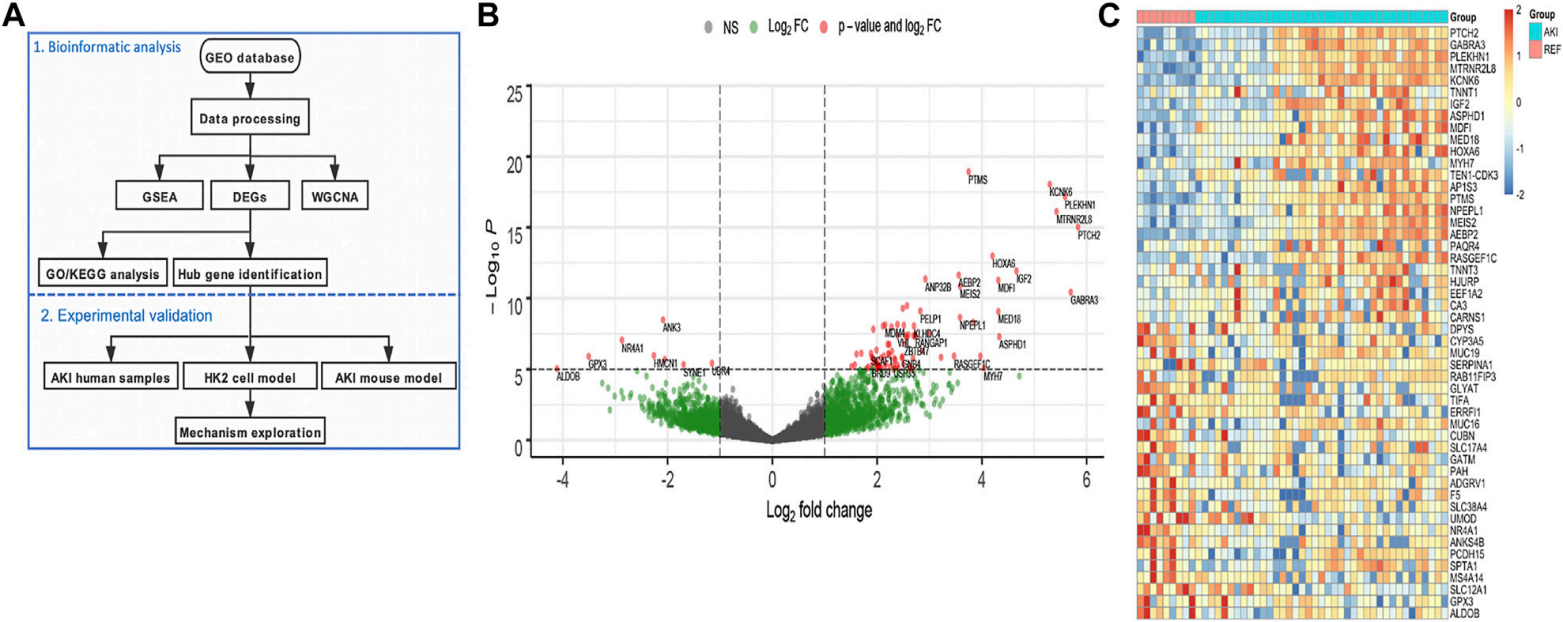
Identification of the differentially expressed genes in AKI patients. **(A)** Flowchart of the bioinformatic analysis and experimental validation. **(B)** Volcano plot of the DEGs between AKI and REF. **(C)** Heat-map of the top upregulated and downregulated 50 DEGs from AKI and REF transcriptome profiles.

To reveal the different gene expression pattern between normal and AKI kidney tissues, 39 native human renal biopsy samples (AKI group) and 9 reference nephrectomies (REF group) of RNA-seq data from GEO were analyzed (Figure 1A). According to a threshold of fold change greater than 2 and *p* value less than 0.05, a total of 1,160 significant DEGs were identified compared to the REF group, including the 747 upregulated and 413 downregulated genes. The distribution of DEGs was visualized using the volcano map (Figure 1B). We then performed heat-map showing the top upregulated and downregulated 50 DEGs (Figure 1C). The different gene expression pattern suggests that various genes may play vital roles in AKI.

### Dysregulated Pathway Identification *via* GSEA

**FIGURE 2:**
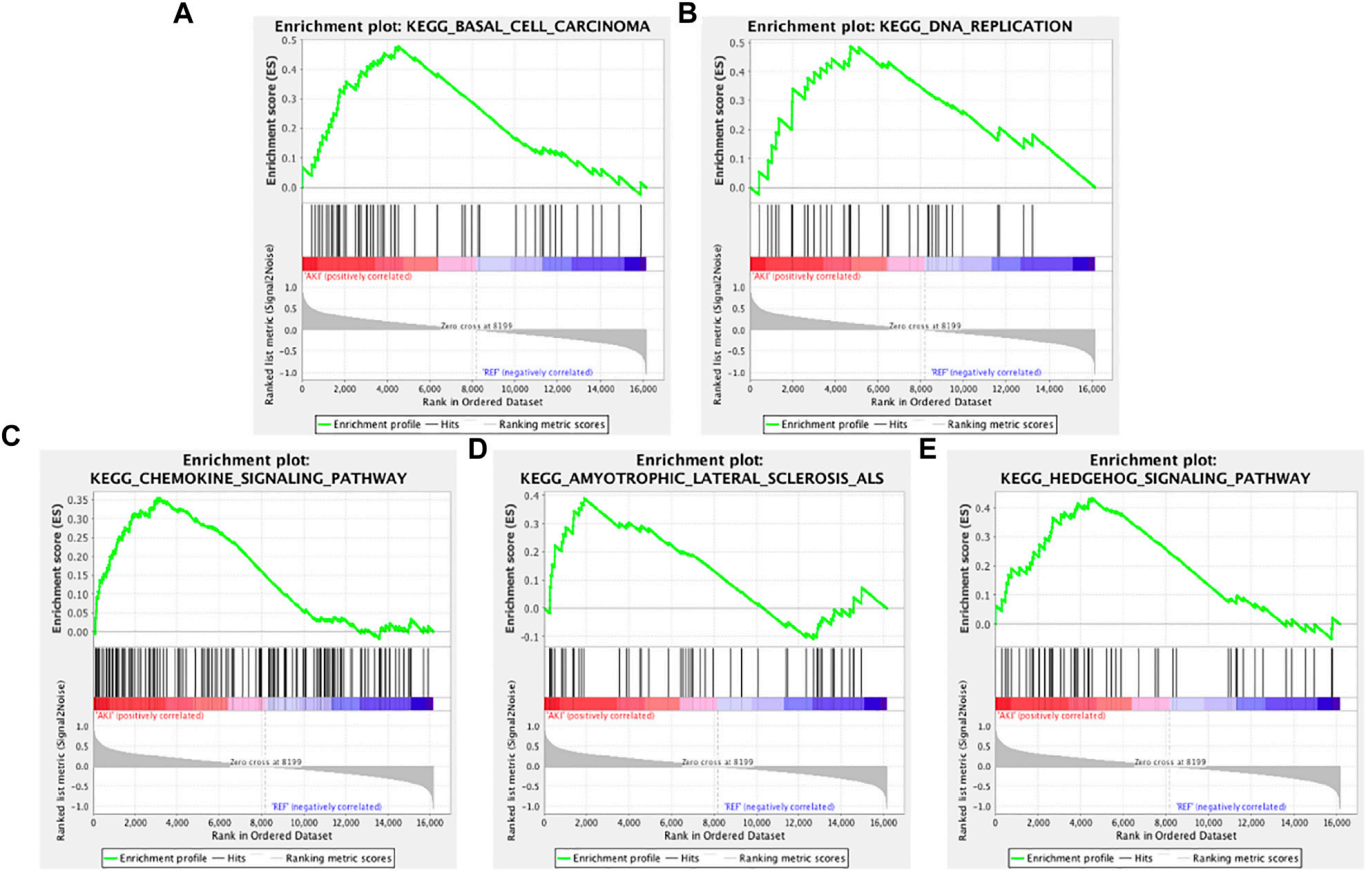
Enrichment plots from GSEA of upregulated pathways in AKI. GSEA results showing the upregulated pathways of **(A)** “basal cell carcinoma,” **(B)** “DNA replication,” **(C)** “chemokine signaling pathway,” **(D)** “amyotrophic lateral sclerosis,” and **(E)** “Hedgehog signaling pathway.”

**FIGURE 3:**
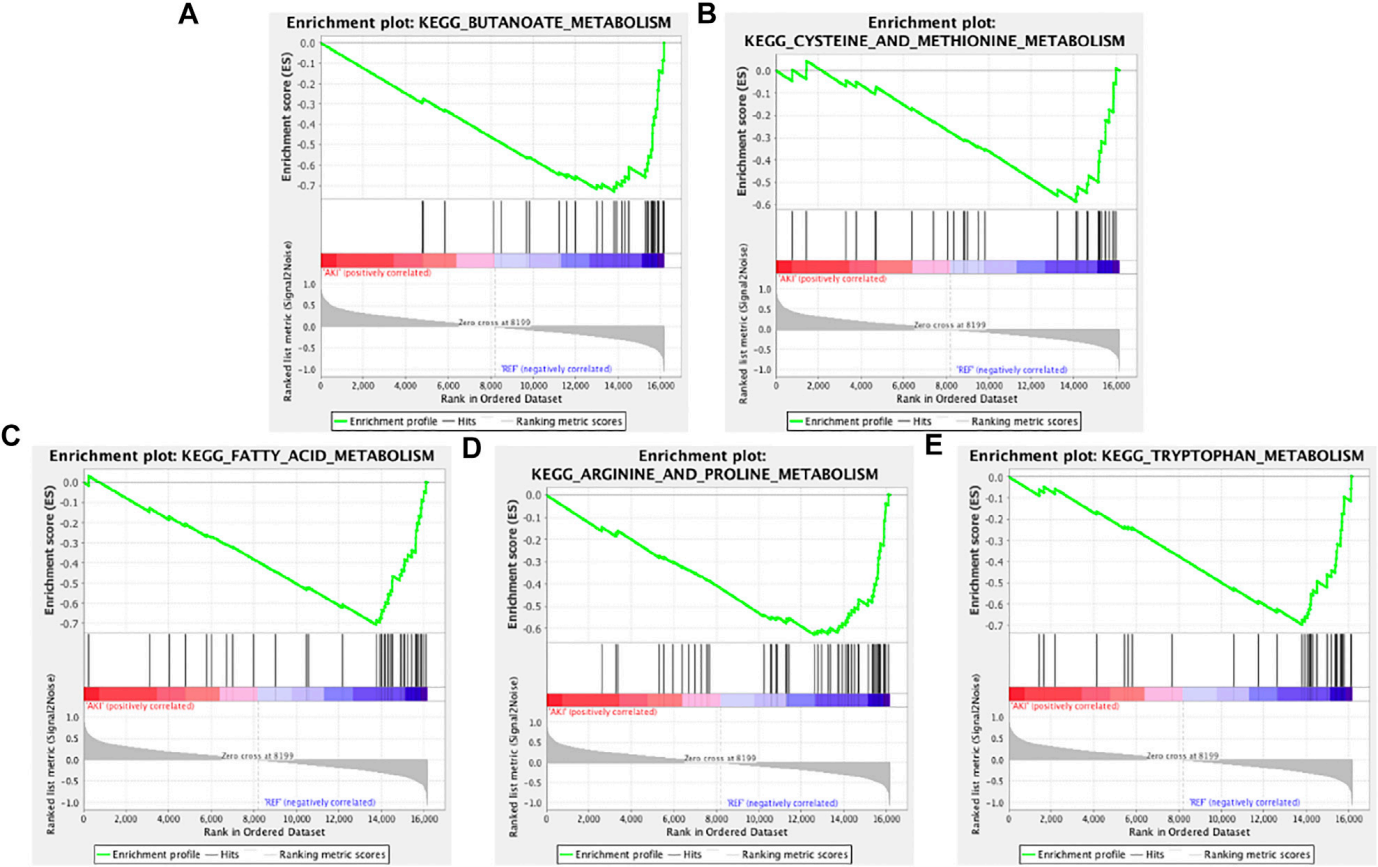
Enrichment plots from GSEA of downregulated pathways in AKI. GSEA results showing the downregulated pathways of **(A)** “butanoate metabolism,” **(B)** “cysteine and methionine metabolism,” **(C)** “fatty acid metabolism,” **(D)** “arginine and proline metabolism,” and **(E)** “tryptophan metabolism.”

To determine whether the Kyoto Encyclopedia of Genes and Genomes (KEGG) pathways were differentially enriched between the AKI and REF groups, GSEA was used to detect the enrichment of KEGG pathways based on NES and *p* values. As illustrated in Figure 2, the significantly upregulated KEGG pathways enriched in the AKI group were “basal cell carcinoma,” “DNA replication,” “chemokine signaling pathway,” “amyotrophic lateral sclerosis,” and “Hedgehog signaling pathway.” Furthermore, the main downregulated KEGG pathways in the AKI group were “butanoate metabolism,” “cysteine and methionine metabolism,” “fatty acid metabolism,” “arginine and proline metabolism,” and “tryptophan metabolism” (Figure 3). These data indicate that many pathways may be involved in the initiation and progression of AKI.

### GO Analysis and PPI Network of the Altered Genes

**FIGURE 4:**
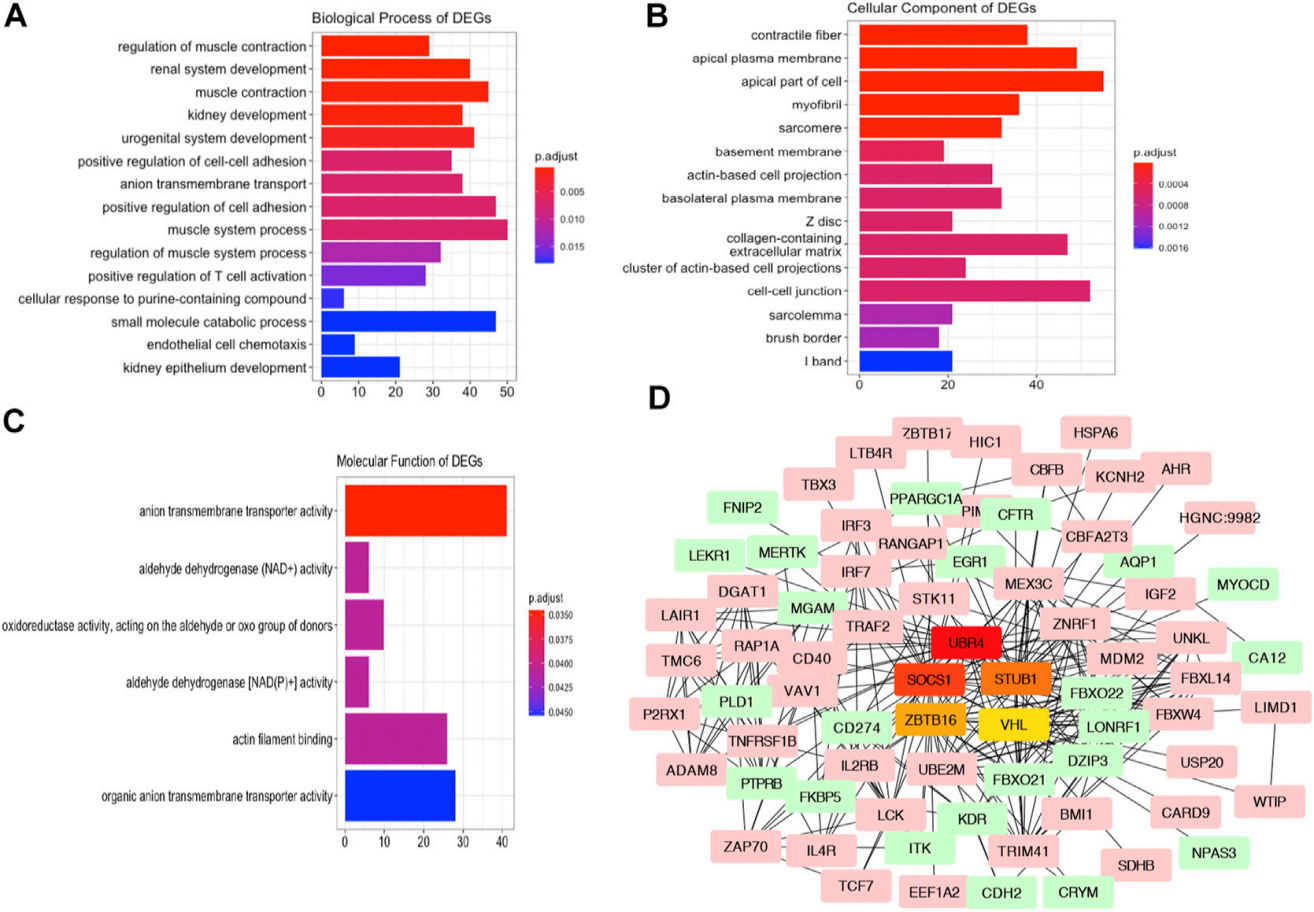
GO Analysis and PPI network of the altered genes. Plots of the enriched GO terms including the BP **(A)**, CC **(B)**, and MF **(C)**. The *x*-axis represents the number of genes in the enriched GO terms. **(D)** Interaction network analysis of DEGs. Pink and green genes represent upregulated and downregulated DEGs. Five hub genes are visualized in the center of the PPI networks.

To investigate the Gene Ontology of DEGs, the upregulated and downregulated DEGs were subjected to GO analysis for the biological process, cellular component, and molecular function categories. As indicated in Figure 4A, the GO analysis of BP suggested that the altered genes were mainly involved in renal system development, regulation of muscle contraction, and kidney development. For the GO CC terms, it was indicated that the DEGs were enriched in the contractile fiber part, sarcomere, and collagen-containing extracellular matrix (Figure 4B). For the MF terms, anion transmembrane transporter activity, aldehyde dehydrogenase (NAD) activity, and aminopeptidase activity were enriched (Figure 4C).

Next, to reveal the correlation of the DEGs and potential hub genes in AKI, we constructed PPI networks of the DEGs using the STRING database. After removing the disconnected nodes, the correlated genes were imported into Cytoscape to visualize the PPI networks. The hub genes in the networks refer to a node connecting many physical connections, which were indicated as vital points in biological networks. As presented in Figure 4D, we screened the top hub genes using the cytoHubba plugin with the ranking method of degree. The upregulated genes *SOCS1*, *STUB1*, and *VHL* and downregulated genes *UBR4* and *ZBTB16* in the AKI group were the top five hub genes identified in the network.

### Network Construction for the AKI and Normal Samples With WGCNA

**FIGURE 5:**
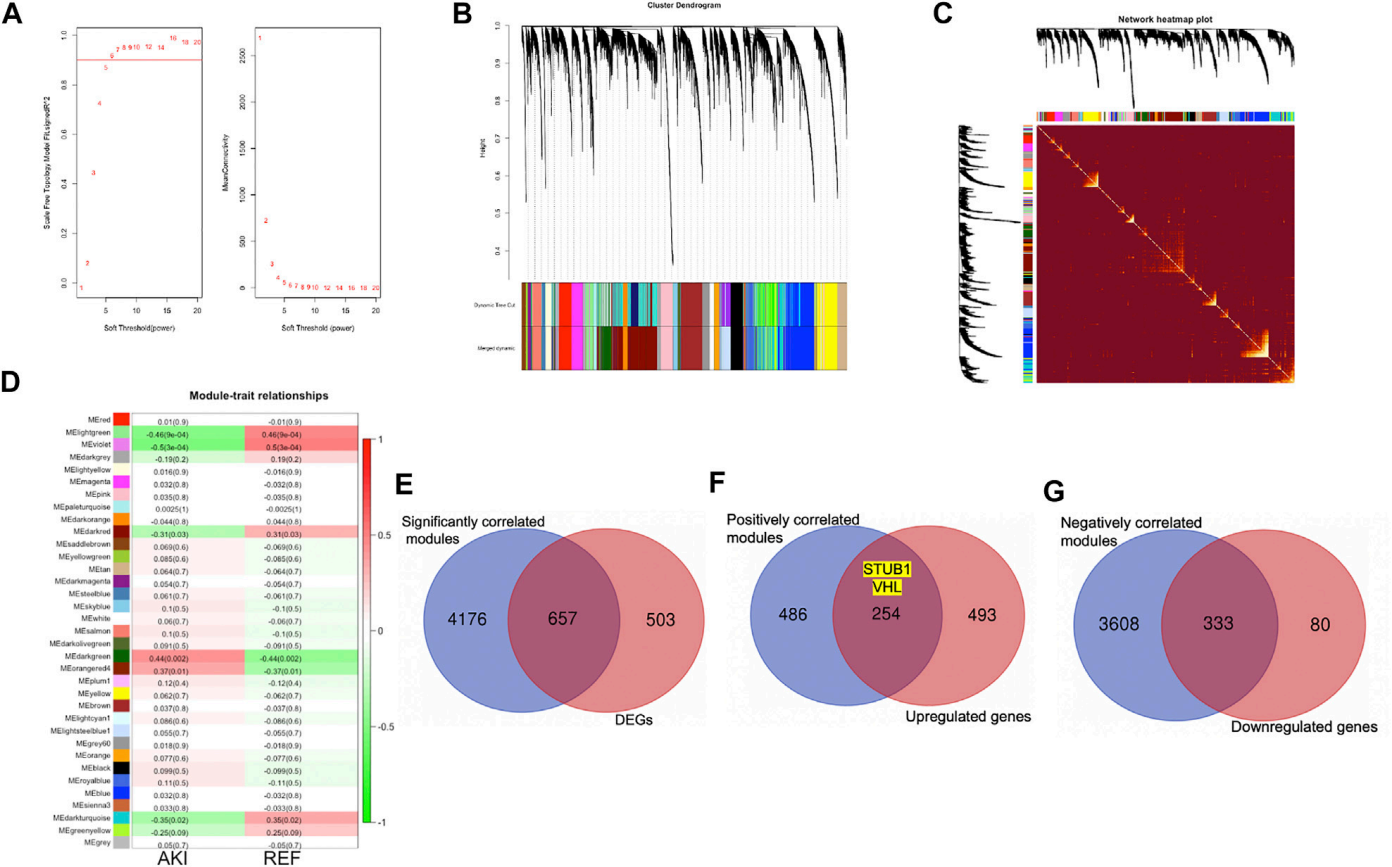
Co-expression network construction by WGCNA. **(A)** Analysis of optimal soft-thresholding power. Analysis of different powers on scale independence **(left)** and mean connectivity **(right)**. **(B)** Cluster dendrogram of 16,128 protein coding genes and 35 modules for AKI and normal sample networks. **(C)** Heat-map of the interactions among co-expression modules. The intensity of yellow represents the interconnectedness among different modules. **(D)** Heatmap of the correlation between the MEs and clinical traits with AKI or REF. **(E)** Overlap of genes identified in both DEGs and significantly correlated modules with AKI. **(F)** Overlap of genes identified in both upregulated genes and positively correlated modules with AKI. **(G)** Overlap of genes identified in both downregulated genes and negatively correlated modules with AKI.

To define the mean connectivity and balanced scale independence of co-expression networks, the soft-thresholding power was carefully considered. As in Figure 5A, the topology network was close to scale-free with a soft-thresholding power value of 8 and a coefficient threshold of 0.9. Subsequently, 35 merged co-expression modules were constructed (Figure 5B). Interaction analysis of the modules showed that the main modules were independent of each other in the network (Figure 5C). Then, the correlation between phenotypes of AKI and REF and module eigengenes (MEs) was determined. The results revealed that the dark-green and orange-red 4 modules were positively correlated with AKI (Figure 5D). Furthermore, we recognized that the light-green, violet, and dark-turquoise modules were negatively correlated with AKI (Figure 5D). Interestingly, we found that 657 genes of the total 1,160 DEGs (56.7%) were in the significantly correlated modules to the illness status of AKI (Figure 5E). Additionally, 254/747 (34%) of the upregulated genes were in the positively correlated modules (Figure 5F), while 333/413 (80.6%) of the downregulated genes were in the negatively correlated modules (Figure 5G). Notably, the upregulated hub genes, *STUB1* and *VHL*, were both in the positively correlated modules with AKI. These results verified the potential molecular mechanism of the upregulated hub genes in relation to AKI.

### Validation of the Upregulated Hub Genes in Human AKI Tissues

**FIGURE 6:**
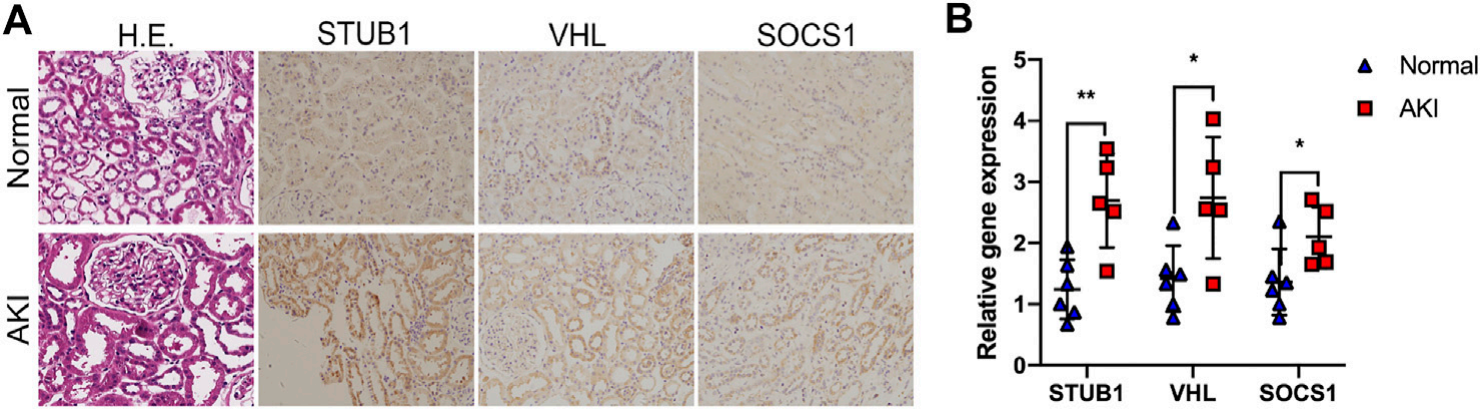
Validation of the upregulated hub genes in human AKI tissues. **(A)** Representative images of HE and IHC staining of the kidney tissues from AKI and normal kidneys, as indicated. Magnification ×200. **(B)** qPCR of STUB1, VHL, and SOCS1 mRNA levels in five AKI tissues and six normal kidney tissues. *p < 0.05, **p < 0.01, compared to control.

To validate the identified hub gene expression in human AKI kidney tissues, we performed IHC staining to examine the gene expression of three upregulated hub genes: *STUB1*, *SOCS1*, and *VHL*. We collected five AKI tissues and six normal kidney tissues from kidney biopsy. The diagnosis of AKI was based on clinical parameters, including serum creatinine, estimated glomerular filtration rate (eGFR), and pathological staining. Histopathologically, kidney biopsies of AKI revealed prominent histopathological alterations, such as renal tubular epithelial cell damage and death and tubular dilatation. A marked loss of brush border in proximal tubules also occurs mainly in tubular–interstitial cells (Figure 6A). IHC staining showed that STUB1, SOCS1, and VHL were mainly located in renal tubular epithelial cells instead of the glomerulus, which indicated their potential roles in AKI (Figure 6A). Moreover, in accordance with the RNA-seq data, the mRNA expression levels of *STUB1*, *SOCS1*, and *VHL* were also upregulated in AKI tissues compared to normal tissues (Figure 6B).

### Validation of the Upregulated Hub Genes in Cisplatin-Induced Acute Kidney Injury Mouse Model

**FIGURE 7:**
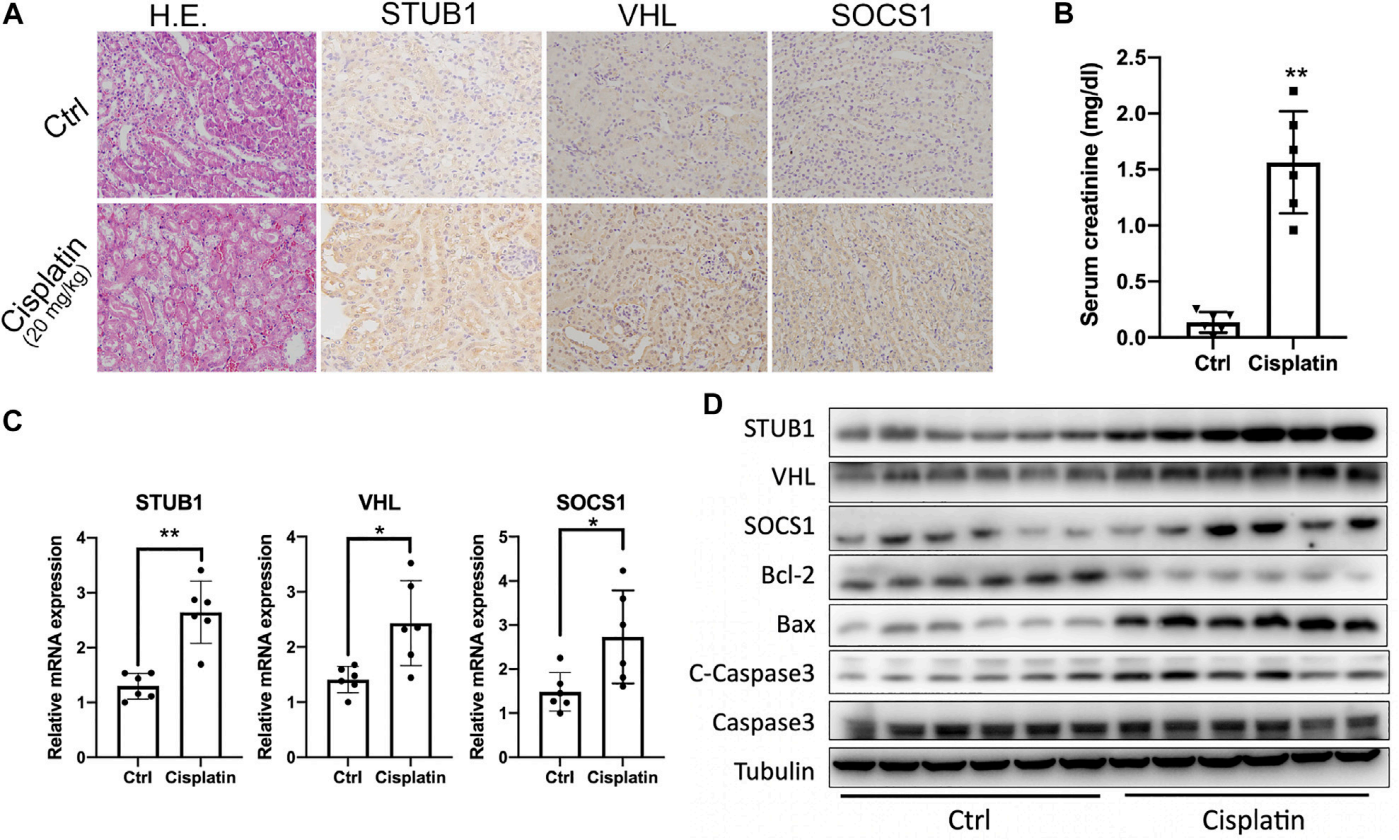
Validation of the upregulated hub genes in the cisplatin-induced AKI mouse model. **(A)** HE and IHC staining of the kidneys from mice treated with control and 20 mg/kg cisplatin. Magnification ×200. **(B)** Serum creatinine levels from mice treated with control and 20 mg/kg cisplatin (*n* = 6). *p < 0.05, **p < 0.01, compared to control. **(C)** qPCR of STUB1, VHL, and SOCS1 mRNA levels in mice treated with control and 20 mg/kg cisplatin (*n* = 6). *p < 0.05, compared to control. ns, no significance. **(D)** Western blot of STUB1, VHL, and SOCS1 and apoptosis proteins in mice treated with control and 20 mg/kg cisplatin (*n* = 6).

We then investigated whether the *STUB1*, *SOCS1*, and *VHL* genes were also upregulated in the mouse AKI model. The “DNA replication” pathway was identified in the AKI group by GSEA, which indicates that those AKI biopsies may be obtained from patients with systemic DNA damage agents, such as platins. Thus, a widely used cisplatin-induced acute kidney injury mouse model was applied to validate our hypothesis. We treated mice with 20 mg/kg body weight of cisplatin and then collected serum and kidney samples for further analysis after 72 h. Histopathologically, cisplatin-treated mice showed swollen cytoplasm and nuclear lysis in affected epithelial cells. The renal tubules showed nuclear alterations, including segmented, dispersed, and condensed heterochromatins, particularly in the cortico-medullary junction (Figure 7A). Moreover, the creatinine levels also significantly increased after cisplatin treatment (Figure 7B). Besides, we found that the cisplatin-treated mice had higher levels of proapoptotic Bax and lower levels of antiapoptotic Bcl-2 than sham mice (Figure 7C). In addition, another key effector of apoptotic activity, cleaved caspase-3, was also significantly increased after cisplatin treatment. Taken together, these data indicated that acute renal injury due to cisplatin was successfully induced.

Subsequently, we determined the expression of the three hub genes in a mouse model. As expected, an evident increase after cisplatin treatment was observed at both the mRNA and protein levels (Figures 7C,D). Cisplatin (20 mg/kg) significantly upregulated STUB1, SOCS1, and VHL. From IHC staining, we also observed that the three hub genes were also mainly expressed in the renal tubules and their expression was consistent with that in human samples (Figure 7A).

### STUB1 Inhibition Promoted Cisplatin-Induced Apoptosis of HK-2 Cells

**FIGURE 8:**
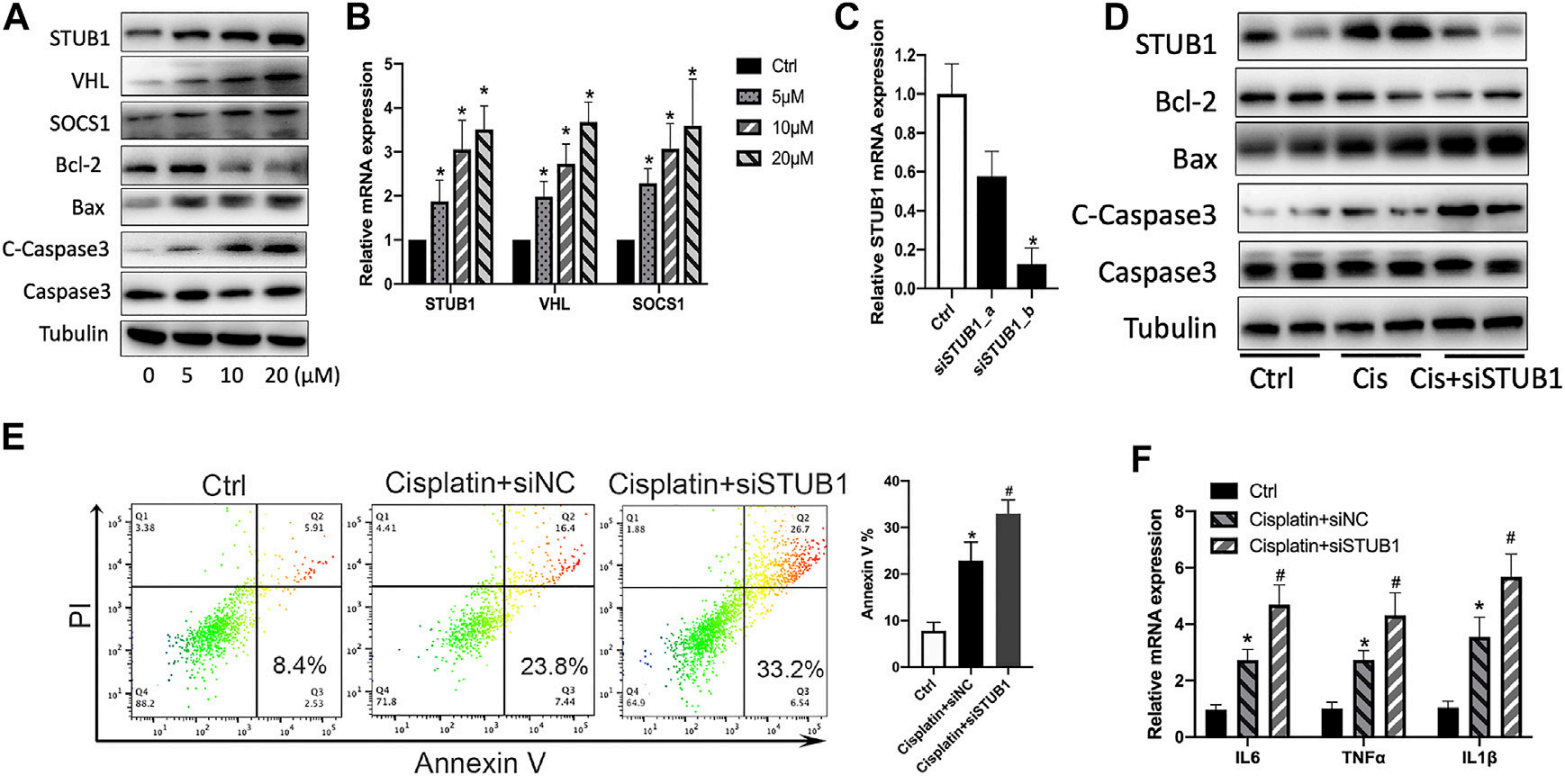
STUB1 inhibition promoted apoptosis of HK-2 cells induced by cisplatin. **(A)** Western blot of STUB1, VHL, and SOCS1 in HK-2 cells treated with 0-5–10–20 μM cisplatin. **(B)** qPCR of STUB1, VHL, and SOCS1 mRNA levels in HK-2 cells treated with 0-5–10–20 μM cisplatin (n = 3). *p < 0.05, compared to control. **(C)** Knockdown efficiency of STUB1 in HK-2 cells by qRT-PCR (*n* = 3). *p < 0.05, compared to control. **(D)** Apoptosis of HK-2 cells treated with 20 μM cisplatin and STUB1 knockdown by western blot analysis. **(E)** Annexin V apoptosis analysis of HK-2 cells treated with 20 μM cisplatin and STUB1 knockdown. *p < 0.05, compared to control; ^#^ *p* < 0.05, compared to the cisplatin + siNC group (*n* = 3). **(F)** Proinflammatory cytokine production of HK-2 cells treated with 20 μM cisplatin and STUB1 knockdown by qRT-PCR analysis (*n* = 3). *p < 0.05, compared to control; ^#^ *p* < 0.05, compared to the cisplatin + siNC group.

To confirm whether STUB1, SOCS1, and VHL increase after cisplatin treatment *in vitro*, we treated the human proximal tubular cell line HK-2 with a variety of doses of cisplatin (5, 10, and 20 μM). We observed an increase of proapoptotic Bax and cleaved caspase-3 and a decrease of antiapoptotic Bcl-2 after cisplatin treatment (Figure 8A). Similar to the *in vivo* results, the mRNA and protein levels of STUB1, SOCS1, and VHL mRNA also increased in a dose-dependent manner (Figures 8A,B).

Based on the observations of STUB1, SOCS1, and VHL in AKI, our results are consistent with previous reports showing that SOCS1 and VHL were increased and involved in AKI (Nakagawa et al., 2002; Schley et al., 2011). However, the roles of STUB1 in kidney injury remain unclear. Thus, we further investigated the biological roles of STUB1 in cisplatin-induced AKI. Because apoptosis of tubular epithelial cells and renal inflammation contribute to cisplatin-induced kidney injury, we evaluated the potential function of STUB1 in the cisplatin-induced apoptosis of HK-2 cells. We specifically knocked down STUB1 using two siRNAs. The knockdown efficiency was validated by qRT-PCR, and a sequence of higher knockdown efficiency was used for further experiments (Figure 8C). Interestingly, 20 μM cisplatin treatment significantly increased the expression of Bax and cleaved caspase-3 and decreased Bcl-2 expression, which was enhanced by STUB1 silencing (Figure 8D). We further determined the effect of STUB1 on HK-2 survival after cisplatin treatment. Annexin V apoptosis assays confirmed that cisplatin induced serious apoptosis of HK-2 cells and that this effect could be promoted by STUB1 knockdown (Figure 8E). Notably, the inflammatory cytokines *IL6*, *TNFα*, and *IL1β* were subsequently upregulated after STUB1 inhibition (Figure 8F). Our findings suggested that STUB1 may serve as a potential target for preventing the initiation and progression of AKI.

## Discussion

AKI is one of the most complicated diseases in kidney diseases, and the molecular mechanism has not been revealed. Thus, studies are urgently needed to identify potential biomarkers and to reveal that molecular mechanisms are important for clinical practice. In the present study, we systematically analyzed the gene expression profiles from 39 native human renal biopsy samples of AKI and 9 reference nephrectomies. A total of 1,160 significant DEGs (747 upregulated and 413 downregulated genes) were identified between the AKI and REF samples. Subsequently, GO and KEGG pathway analyses were performed to identify the enriched molecular functions and pathways. To reveal the molecular function of the identified DEGs, a hub gene analysis was performed to reveal the potential genes involved in AKI. Upregulated *STUB1*, *VHL*, and *SOCS1* and downregulated *UBR4* and *ZBTB16* were identified as the hub genes. WGCNA confirmed most of the DEGs were involved in significantly correlated modules with AKI, especially two upregulated hub genes, *STUB1* and *VHL*. We then validated the expression of the three upregulated genes *STUB1*, *VHL*, and *SOCS1* in human AKI, mouse AKI, and cell line models. Additionally, we demonstrated that STUB1 inhibition could mitigate the apoptosis and inflammation of human proximal tubular cells *in vitro*.

Various etiologies contribute to the initiation of AKI, such as suffering from ischemia, toxic agents, decreased kidney perfusion, and inflammation (Ali et al., 2007). Thus, the pathophysiology and molecular mechanisms vary with these conditions. GSEA revealed that several pathways were involved in AKI, including “DNA replication,” “chemokine signaling pathway,” and “Hedgehog signaling pathway.” For patients with carcinomas, chemotherapy is one of the main therapeutics, and it includes DNA damage agents, such as platins. Thus, “DNA replication” indicates that those biopsies may be obtained from patients with systemic chemotherapy. Therefore, we used cisplatin-induced mouse and cell line models to validate the hub gene expression and explore the molecular function. This pathway is involved in many DNA damage repair genes, including *POLD4*, *DNA2*, *POLE4*, and *MCM4*. Chemokines were also involved in AKI. In animal models induced by folic acid overdose, cisplatin, or unilateral ureteral obstruction, the chemokine CCL20 was upregulated and had a nephroprotective role by facilitating repair and decreasing tissue injury (González-Guerrero et al., 2018). In AKI with sterile inflammation, B cells produce CCL7 to recruit neutrophils and monocytes to the injured kidney (Inaba et al., 2020). In our analysis, *CCL5*, *CCL19*, *CCL21*, *CXCR5*, and *CXCL2* were upregulated in AKI, which was consistent with that reported in previous studies (Sanz et al., 2010; Winterberg et al., 2013; Wohlfahrtova et al., 2015; Sobbe et al., 2020). Moreover, Hedgehog signaling was reported to be upregulated and protected tubular cells in various AKI models, such as ischemia/reperfusion (Zhuo et al., 2019) and obstructive kidney injury (Rauhauser et al., 2015). For the downregulated pathways in the AKI group, the metabolism of amino acids and fatty acid pathways, such as “butanoate metabolism,” “cysteine and methionine metabolism,” and “fatty acid metabolism,” were downregulated, indicating the impaired renal function. Consistently, the GO analysis revealed the enriched molecular functions of anion transmembrane transporter activity, aldehyde dehydrogenase (NAD) activity, and aminopeptidase activity. The myofunction of amino acid metabolism and small molecule transport was identical to the pathophysiology of AKI (Karmin and Siow, 2018; Perazella, 2019).

Upregulated hub gene identification may provide potential molecules responsible for the initiation and progression of AKI. *VHL* is upregulated and belongs to a positively correlated module, the orange-red 4 module, with AKI. Renal hypoxia occurs in AKI under many conditions and is mediated by hypoxia-inducible factor (HIF). Stabilizing HIF before ischemia attenuates tissue injury, while degrading HIF by von Hippel–Lindau (VHL) protein contributes to AKI under normoxia. Deficiency of VHL in the thick ascending limb attenuated proximal tubular injury following ischemia–reperfusion (Schley et al., 2011). Additionally, VHL deficiency was also reported to protect against rhabdomyolysis-induced AKI by activating HIF in nephron segments (Fähling et al., 2013). SOCS1 is a suppressor of cytokine signaling and a negative regulator of the NF-κB pathway (Nakagawa et al., 2002). Suppression of SOCS1 in macrophages by treatment with miR-19b-3p from tubular epithelial cell exosomes mitigated tubulointerstitial inflammation (Lv et al., 2020). In addition, as an inhibitor of the JAK2/STAT1 pathway, the adenosine monophosphate protein kinase (AMPK) activator ameliorated cisplatin-induced acute tubular injury by suppressing the JAK2/STAT1 pathway and upregulating SOCS1 (Tsogbadrakh et al., 2019). In our present study, the clinical renal biopsies, a cisplatin-induced mouse kidney injury model, and an *in vitro* model all confirmed our finding of the upregulated hub genes. Notably, these genes were mainly expressed in renal tubular epithelial cells instead of the glomerulus, which is consistent with the pathophysiology of AKI. These results were consistent with previous reports that VHL and SOCS1 were upregulated in AKI.

However, E3 ubiquitin ligase STUB1, which is an upregulated hub gene, is less known in AKI. *STUB1* also belongs to a positively correlated module, the dark-green module, with AKI. To determine the roles of STUB1 in AKI, a widely used animal model of cisplatin-induced AKI (Faubel et al., 2004; Ozkok et al., 2016) was applied to test our findings. STUB1 was upregulated in the cisplatin-induced AKI mouse model, and cisplatin treatment upregulated STUB1 expression in a dose-dependent manner in HK-2 cells. Furthermore, inhibition of STUB1 by specific siRNAs increased HK-2 cell apoptosis and proinflammatory production, which indicates the role of STUB1 in AKI, particularly in cisplatin-induced AKI. However, the mechanism by which STUB1 attenuates renal tubular epithelial cell apoptosis and inflammation remains unclear. In NIK (NF-κB–inducing kinase)-mediated liver injury, liver-specific overexpression of STUB1 reversed hepatic inflammation by ubiquitination and degradation of NIK (Jiang et al., 2015). In colorectal cancer (Wang et al., 2014), breast cancer (Jang et al., 2011), and gastric cancer (Liu et al., 2015), STUB1 functioned as a tumor suppressor and impaired the malignancy of colorectal, breast, and gastric cancer cells by inactivating NF-κB signaling. However, it has been reported that STUB1 catalyzed CARMA1 ubiquitination and then activated NF-κB, and silencing of STUB1 diminished canonical NF-κB activation and IL2 production (Wang et al., 2013). Based on the findings for STUB1, we hypothesize that NF-κB may serve as a target of STUB1 and that the upregulation of STUB1 is associated with the feedback of NF-κB activation. Thus, further studies are needed to reveal the possible mechanisms of the STUB1-NF-κB axis in AKI.

## Conclusion

The results of this study indicate that a number of genes and biological pathways are significantly altered in AKI. Bioinformatic analysis showed that *STUB1*, *VHL*, and *SOCS1* were upregulated, while *UBR4* and *ZBTB16* were downregulated in AKI. *STUB1*, *VHL*, and *SOCS1* were further verified to be upregulated in human AKI tissues and a cisplatin-induced AKI mouse model. Moreover, STUB1 inhibition promoted cisplatin-induced renal tubular epithelial cell apoptosis and inflammation. Our data indicated that STUB1 may protect against renal injury and act as a potential therapeutic target for kidney diseases.

## Data Availability

Publicly available datasets were analyzed in this study. This data can be found here: GEO dataset (GSE139061).
